# Editorial Comment: Novel Treatment for Premature Ejaculation in the Light of Currently Used Therapies: A Review

**DOI:** 10.1590/S1677-5538.IBJU.2020.04.10

**Published:** 2020-05-20

**Authors:** Valter Javaroni

**Affiliations:** 1 Departamento de Andrologia Hospital Federal do Andaraí Rio de JaneiroRJ Brasil Departamento de Andrologia, Hospital Federal do Andaraí, Rio de Janeiro, RJ, Brasil

Porst H ^1^, Burri A ^2^

^1^ European Institute for Sexual Health (EISH), Hamburg, Germany; ^2^ European Institute for Sexual Health (EISH), Hamburg, Germany

Sex Med Rev. 2019 Jan;7(1):129-140

**DOI: 10.1016/j.sxmr.2018.05.001 | ACCESS: 10.1016/j.sxmr.2018.05.001**

## COMMENT

In this recent review, Dr. Hartmut Porst and Andrea Burri pointed the actual situation of Premature Ejaculation (PE) arguing that there is a gap between what doctors are prescribing and what patients expect from treatment.

The only so far officially approved medication – dapoxetine - is characterized by high discontinuation rates of up to 90%, mostly because of high side effects, cost issues, efficacy below expectations, and the need for scheduling sexual intercourse.

The authors discussed advantages and disadvantages of currently available off-label and officially approved treatment options and presented the dose-metered lidocaine-prilocaine spray (Fortacin™), the first topical treatment to be officially approved in Europe for the treatment of primary PE in adult men.

The use of drugs that selectively reduce penile sensitization or which modify the afferent-efferent reflex could provide effective therapy for PE, as has been shown with the off-label use of topical desensitizing creams ( 1 ) that represents the oldest form of pharmacotherapy in PE (1943).

There are many studies ( 2 , 3 ) demonstrating safety and efficacy of this lidocaine-prilocaine spray (first known as TEMPE and also PSD502) that seems to have some advantages from creams, since its special galenic properties generates a stable mixture which can be readily absorbed through the glans penis mucous membrane, but not through normal keratinized skin, maximizing the extent of neural blockage and minimizing the onset of numbness ( 4 ).

Fortacin ™ was officially approved for use in the European Union in 2013 and finally launched in the United Kingdom in November 2016. This lidocaine-prilocaine spray, with all pharmacological advantages and well conducted trials, has not yet reached a significant first-line therapy status both for the physicians and the patients in PE. There are no comments from the authors to contradict or explain this fact.
